# Oral sorafenib-loaded microemulsion for breast cancer: evidences from the in-vitro evaluations and pharmacokinetic studies

**DOI:** 10.1038/s41598-022-17333-6

**Published:** 2022-08-12

**Authors:** Nishtha Chaurawal, Charu Misra, Harshita Abul Barkat, Reena Jatyan, Deepak Chitkara, Md. Abul Barkat, Teenu Sharma, Bhupinder Singh, Kaisar Raza

**Affiliations:** 1 Department of Pharmacy, School of Chemical Sciences and Pharmacy, Central University of Rajasthan, Bandarsindri, Ajmer, Rajasthan 305817 India; 2 Department of Pharmaceutics, College of Pharmacy, University of Hafr Al-Batin, Al Jamiah, Hafr Al Batin, 39524 Saudi Arabia; 3 Department of Pharmacy, Birla Institute of Technology and Science (BITS)-Pilani, Pilani Campus, Pilani, Vidya Vihar, Rajasthan 333031 India; 4 University Institute of Pharmaceutical Sciences, Panjab University, Chandigarh, 160014 India; 5 Department of Pharmacy, Chandigarh College of Pharmacy, Landran, Punjab 140307 India

**Keywords:** Nanoparticles, Breast cancer

## Abstract

Sorafenib tosylate (SFB) is a multikinase inhibitor that inhibits tumour growth and proliferation for the management of breast cancer but is also associated with issues like toxicity and drug resistance. Also, being a biopharmaceutical class II (BCS II) drug, its oral bioavailability is the other challenge. Henceforth, this report intended to encapsulate SFB into a biocompatible carrier with biodegradable components, i.e., phospholipid. The microemulsion of the SFB was prepared and characterized for the surface charge, morphology, micromeritics and drug release studies. The cell viability assay was performed on 4T1 cell lines and inferred that the IC_50_ value of sorafenib-loaded microemulsion (SFB-loaded ME) was enhanced compared to the naïve SFB at the concentrations of about 0.75 µM. More drug was available for the pharmacological response, as the protein binding was notably decreased, and the drug from the developed carriers was released in a controlled manner. Furthermore, the pharmacokinetic studies established that the developed nanocarrier was suitable for the oral administration of a drug by substantially enhancing the bioavailability of the drug to that of the free SFB. The results bring forth the preliminary evidence for the future scope of SFB as a successful therapeutic entity in its nano-form for effective and safer cancer chemotherapy via the oral route.

## Introduction

According to WHO, 9.6 million people died worldwide in the last 10 years because of cancer^1^. There are various types of cancer, like liver cancer, skin cancer, prostate cancer, colon cancer, leukaemia. The most common cancer is breast cancer, which is a major cause of worldwide deaths due to cancer^2^. The etiology behind the incidence of breast cancer are hormonal factors, environmental factors and lifestyle changes. Breast cancer is mainly associated with the complex genetic behaviour of the person^3^.

**Figure 1:**
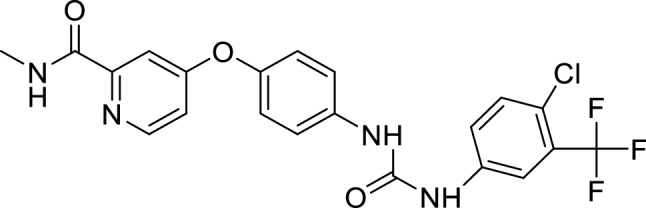
Structure of SFB.

Protein kinase plays a vital role in regulating cellular functions like metabolism, differentiation, signal transduction, survival, and programmed cell death^4^. Sorafenib tosylate (SFB), a multikinase inhibitor drug, was developed by the Bayer and Onyx companies as BAY 43-9006 in 2001. Its patent was issued in 2004 from the United States Patent and Trademark Office (USPTO). SFB possesses anti-proliferative and anti-angiogenic effects^5^. The structure of SFB is shown in Fig. 1**.** SFB was clinically approved for treating cancer in December 2006 by US-FDA and in August 2007 by the central drug standard control organisation (CDSCO)^6^. It is used to treat hepatocellular carcinoma, renal carcinoma, breast cancer, thyroid cancer, and prostate cancer. The mode of action of SFB is the serine/threonine kinases c-Raf (Raf-1) inhibition and B-Raf. SFB also inhibits the tyrosine kinase receptor Flt-3 and RET, involved in the pathogenesis of breast cancer^7^. Various clinical trials to identify its therapeutic efficacy on breast cancer are in different phases^8^. Despite its high therapeutic effectiveness in cancer management, it also possesses various side effects on oral administration such as diarrhoea (30%), fatigue (18%), hypertension (8–16%), pancreatitis (< 1%). SFB is also linked with dermatological side effects such as seborrheic dermatitis, hand-foot skin reaction, alopecia, rash, stomatitis, and erythema. The dermatological side effects are the most commonly reported side effects of SFB. It also possesses pharmacokinetic related challenges like less oral bioavailability (< 30%), reduced half-life (25–48 h), a plasma level of 7 days and extensive first-pass metabolism. It also exhibits poor water solubility, a BCS class II drug^9^. All these challenges are associated with the SFB oral administration provide a scope for the researchers' to focus on this aspect. In recent past, attempts have been made to develop various nanoformulations of SFB such as lipid polymer hybrid nanoparticles^10^, liposomes^11^, self-emulsifying drug delivery systems^12^, cyclodextrin-modified silicon nanoparticles^13^, nanogels^14^, diatomite nanoparticles^15^, nano colloidal carrier^16^, and pullulan nanoparticles^17^. Most of the developed nanocarriers are non-oral, and very few studies on the oral delivery of SFB employing nanocarriers are reported.

Microemulsions (ME) are biocompatible and thermodynamically stable drug delivery cargoes consisting of aqueous and organic phases stabilized by emulsifiers or surfactants. MEs have proven their efficiency in drug delivery through promising outcomes in drug loading enhancement, controlled drug release, increased scalability, and reduced toxicity^18^. Microemulsions are not nanoemulsions, though their droplet size is in the range of 10–300 nm, the difference lies in the thermodynamic stability, methodology and composition^19–21^. Previously, several research groups have explored the efficacy of ME based formulations in the oral delivery of anticancer drugs^22–24^. Inclusion of phospholipids are known to improve the safety and efficacy of the therapeutic moiety, when incorporated in the nanocarriers by one or other means^25^.

Considering the challenges in oral delivery of SFB, a ME based formulation was optimized and developed employing phospholipid as one of the major components. Pre-clinical studies including in-vitro and in-vivo experiments were performed to evaluate the therapeutic efficacy of SFB-loaded ME. The developed formulation is novel as the ME of SFB is not reported till date.

## Materials and methods

### Chemicals and instruments

Sorafenib tosylate was provided as a generous gift from the M/s Cipla Pvt. Ltd., Mumbai, India. Phospholipon 90G was also a gift sample from Lipoid Gembh, Germany via Chemet, New Delhi. Soranib tablets were purchased from local pharmacy store, manufactured by M/s Cipla Ltd., Mumbai, India. Tween 80, Tween 60, Tween 40, Tween 20, methanol, and dialysis membrane was purchased by M/s Fisher Scientific [Pvt] Ltd, Mumbai, India, M/s SDFCL Chem limited, Mumbai, India, and M/s Himedia laboratories [Pvt] Ltd, Mumbai, India, respectively**.** Isopropyl myristate was purchased from M/s Kempaphore Pvt. Ltd., Mumbai, India. The 4T1 cell lines were provided as a gift sample from Dr. Deepak Chitkara, Assistant Professor, BITS, Pilani, India. Coumarin-6, 4′,6-diamidino-2-phenylindole (DAPI) and Fluorescein IsoThioCyanate (FITC) were purchased from M/s TCI Chemicals Pvt. Ltd., Chennai, M/s Sigma Aldrich, USA and M/s SRL Research Laboratories Pvt. Ltd., Chennai respectively. The Dulbecco’s modified eagle medium and Fetal bovine serum were purchased from M/s Thermo Fisher Scientific, USA. The MTT (3-(4,5-diemthylthiazol-2-yl)-2,5-diphenyltetrazolium bromide) (Catalogue no. 298931) was purchased by M/s Sigma Aldrich, USA. The phosphate buffer saline and dimethyl sulfoxide were purchased from M/s Hi-Media Laboratories, India. The solvents acetonitrile, n-octanol, methanol and water used in the study were of HPLC grade and purchased from M/s Spectrochem [Pvt] Ltd, Mumbai, India. All the material reagents and chemicals utilized in this research were pure and of analytical grade and were used as such.

### Instrumentation

The types of equipment used in this report are ultra-high performance liquid chromatography (UHPLC, Agilent 1290 Infinity LC System, USA) for the drug quantification at various preformulation stages. The particle size, size distribution, polydispersity index (PDI) and zeta potential of the SFB-loaded ME and blank ME were carried out through Malvern Zetasizer (M/s Malvern, Worcestershire, UK). The shape and surface morphology of the ME were observed using transmission electron microscopy (TEM, M/s FEI Tecnai, Europe). The Fourier-transform infrared spectroscopy (FT-IR, M/s Brukers Alpha II, USA) was performed for the drug's structural analysis and physical compatibility of tween 80, IPM and PL 90G over a wavenumber of 4000–400 cm^−1^. The ELISA plate reader (Biotek Epoch Microplate Reader) was used for the determination of percent cell viability.

### Physicochemical characterization of SFB

The SFB was examined for partition coefficient (log P) by shake flask method using pre-saturated n-octanol and water mixture^26^. Melting point (M.P) was determined using M-560 BUCHI Machine, Switzerland, as per the standard protocol.

### Construction of pseudo ternary phase diagram

On the basis of solubility of SFB in surfactant and Isopropyl myristate (IPM), IPM was selected as the oil phase^27^. Tween 80 (T80), Tween 60 (T60), Tween 40 (T40) and Tween 20 (T20) were employed as the surfactants, whereas phospholipid 90G (PL 90G) and ethanol were utilized as the cosurfactants. The distilled water was used as the aqueous phase to design phase diagrams. The eight different groups for the phase diagram were formed considering oil, surfactants and cosurfactants. As mentioned in Table 1, 2.75: 1 and 1: 1 were selected as the ratio of surfactant and cosurfactant (S_mix_ ratios) for each surfactant. The ratio of cosurfactants i.e., PL 90G and ethanol was maintained at 1: 10 throughout the experiment to construct the pseudo ternary phase diagram. The ratio of S_mix_ and aqueous phase was varied from 1: 9–9: 1, which were titrated with oil until they reached the turbidity. Likewise, the ratio of S_mix_ and oil was varied in between 1:9 to 9:1 followed by titration with an aqueous phase until the turbidity appeared. The pseudo ternary phase diagram was constructed for obtaining the ME physical state by marking one axis as oil phase, the other as aqueous phase and the third one as a fixed S_mix_ ratio. Evaluating the diagram through visual inspection, the area which corresponds to the formation of clear and transparent formulation was identified as the ME region in the pseudo ternary phase diagram^19^.

**Table 1:** The oil, surfactant, and cosurfactant group in various predetermined combinations.

Group	Oil	Surfactant	The ratio of surfactant: cosurfactant (S_mix_)
A	IPM	T20	1:1
B	IPM	T20	2.7:1
C	IPM	T40	1:1
D	IPM	T40	2.7:1
E	IPM	T60	1:1
F	IPM	T60	2.7:1
G	IPM	T80	1:1
H	IPM	T80	2.7:1

### Selection of formulation based on phase diagram

After constructing the phase diagram, the minimum quantity of surfactant to a higher fixed value was chosen to prepare different formulations. The selected ratio was supposed to represent the whole pseudo ternary phase diagram.

### Preparation of microemulsion

**Figure 2:**
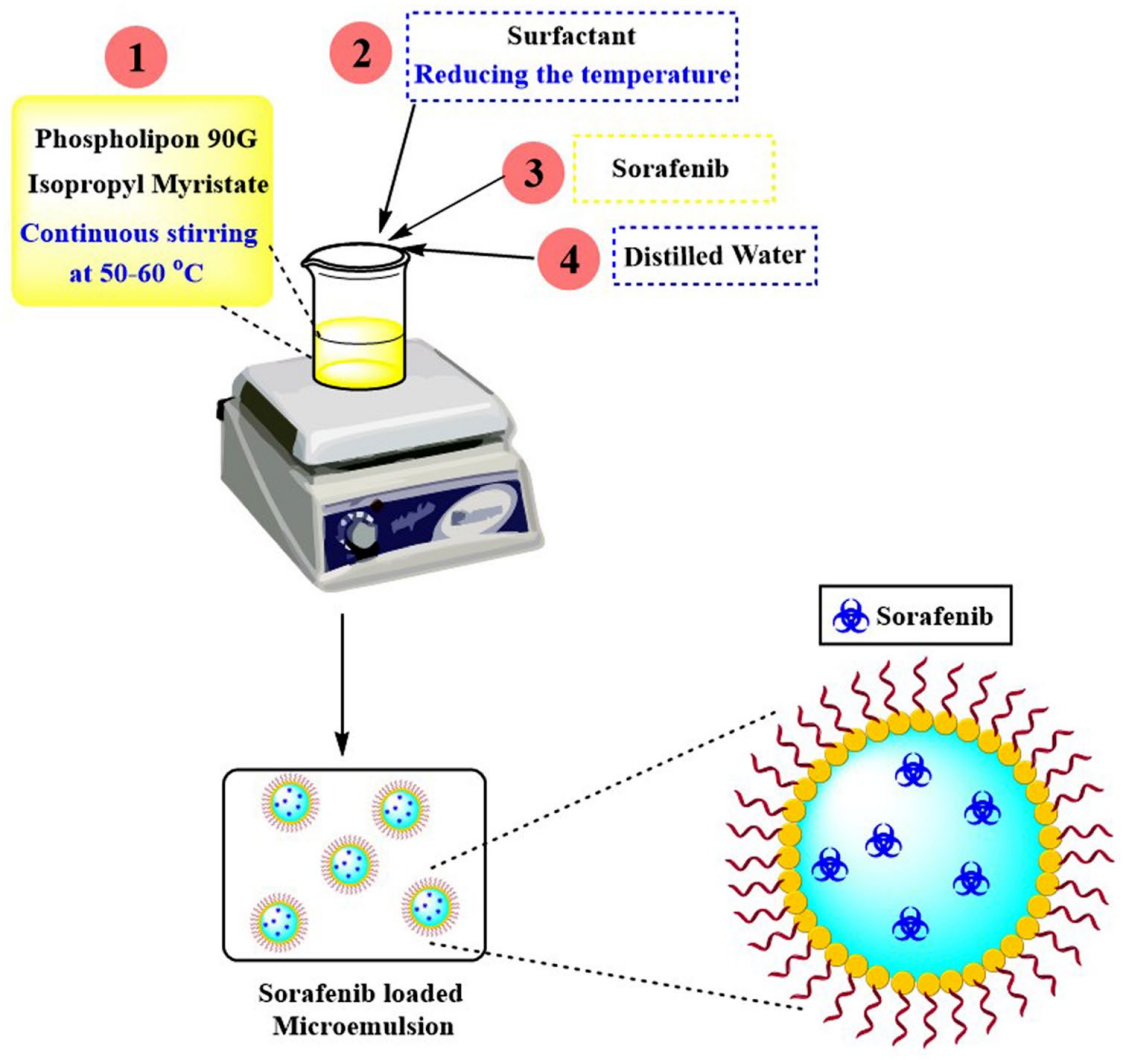
Schematic representation for the preparation of SFB-loaded microemulsion.

The ME was developed based on the selected ME areas in the ternary phase diagram at different ratios of components. All MEs were prepared to employ the emulsification method. Firstly, the PL 90G was mixed with IPM at a temperature of 50–60 °C with continuous stirring (700 rpm for 10 min). Then, the surfactant was poured to the mixture while reducing the system's temperature. After that, SFB was incorporated into the system with gentle stirring, and the required amount of aqueous phase was poured dropwise in the above ME with continuous stirring. A clear and transparent ME, having yellowish colour, was obtained upon the addition of ethanol. A pictorial representation of the preparation method has been described in Fig. 2.

### Drug content

To determine the drug content in the prepared SF-loaded ME, SFB was excerpted in methanol and quantified using UHPLC. Blank ME without drug was used as the blank formulation.

### Percent drug loading (DL) and drug entrapment (EE) studies

EE and DL, the formulation studies were performed using the dialysis method. The SFB-loaded ME (eq. to 1 mg of SFB) was weighed and packed carefully in dialysis bag. The dialysis bag was then placed on magnetic stirrer for continuous stirring in 100 mL beaker containing 50 mL of methanol for 2 h. After 2 h, the 1mL sample was withdrawn from the beaker, and the sample was quantified for SFB using UHPLC^28^. Further, the percent drug entrapment and percent drug loading were calculated using the following formula^29^.

$$\%Entrapment \; Efficiency= \frac{Amount \; of \; drug \; \left(\text{mg}\right)-Amount \; of \; unentraped \; drug \; (\text{mg})}{Amount \; of \; drug\; (\text{mg})}*100$$

$$\% Drug \; loading=\frac{\left(theoretical \; drug \;\left(\text{mg}\right)-amount \; of \; unentraped \; drug \; \left(\text{mg}\right)\right)}{amount \; of \; carrier \;used \; (\text{mg})}*100$$

The drug amounts in the dialysis bags were also quantified to confirm the results.

### In-vitro drug release and release kinetics

Drug release and drug kinetic studies were performed by placing free SFB (1 mg), SFB tablet (marketed formulation; batch no. GJ10330) (equivalent to 1 mg of free SFB) and SFB-loaded ME (equivalent to 1 mg of free SFB) in dialysis bags. In brief, the SFB and SFB tablet suspension and the ME were packed in separate dialysis bags. Each bag was suspended separately in 50 mL of 0.1 N HCl (pH 1.2) containing 1% Tween 80 for the first two hours with continuous stirring and then dipped individually into the 50 mL of phosphate buffer saline (PBS) 7.4 containing 1% Tween 80 for a total of 24 h^30^. The 2 mL samples were withdrawn from each beaker at regular time intervals and adequately maintained the sink conditions. Then cumulative drug release was determined using the UHPLC^31^. The data of percentage drug release were fitted on all the release models such as first-order, zero-order, and Higuchi model with their details enlisted in Table 2^32^.

**Table 2:** Details of various drug release models.

Release model	Parameter on x-axis	Parameter on y-axis	Equation
Zero-order	Time	% Drug release	C_t_ = C_0_ + K_0_t
First-order	Time	Log % drug release	Log C_t_ = log C_0_ + K_t_t/2.303
Higuchi	The square root of time	% Drug release	C_t_ = A√D (2C_0_ – C_s_) C_s_t

where, C_t_ = amount of drug dissolved in time t, C_0_ = initial amount of drug in the solution, K_0_ = zero order release constant, K_t_ = first order release constant, C_s_ = drug solubility in the matrix media, D = diffusivity of drug molecules in the matrix, M_t_/M_∞_ = fraction of drug released at time t, K_p_ = release rate constant, n = release exponent.

### Protein binding studies

The PBS of pH 7.4 was prepared and mixed with human serum albumin (HSA), 4% w/v. The pure drug (SFB, 2.5 mg) was dispersed in PBS 7.4 (1 mL) and SFB-loaded ME containing an equal amount of drug were packed in dialysis membrane bags. To the previously prepared HSA dispersion (4% w/v), the dialysis bags containing different samples were immersed for 12 h with constant stirring. The samples were withdrawn after 12 h followed by centrifugation at 14,000 rpm (21.036×*g*) at 4 °C. After centrifugation, the supernatants were removed, mixed with an equal amount of acetonitrile (ACN) and filtered through a 0.22 µm filter. The samples were then analysed through UHPLC at the respective wavelength for the quantification of free SFB^33,34^.

### In-vitro cytotoxicity assay

The cytotoxic effects of the developed nano-formulation (SFB-loaded ME) were evaluated against 4T1 breast cancer cells and were compared to that of the blank formulation and free SFB. The method was performed by the relevant guidelines and regulations. 4T1 cells (mouse mammary carcinoma) were cultured in culture plates of 96 wells using DMEM culture media. Different concentrations of SFB, blank formulation, and SFB-loaded ME (0.25–50 µM) were prepared separately using the same culture media. The prepared samples were transferred into the 96-well culture plates containing 4T1 cells followed by incubation of 24 h at 37 °C. Further, 10 μL of MTT solution was added, and the 4T1 cell culture plates were re-incubated for an additional 4 h. The purple-coloured formazan crystals were dissolved in 200 μL of DMSO. Using an ELISA plate reader, the samples were analysed for optical density at λmax of 570 nm. Percentage cell viability was determined in comparison to untreated cells. The results obtained from optical density values were utilised to calculate the percentage cell viability. The samples were also analysed for microscopic observation using an optical microscope^35^.

### In-vitro cellular uptake studies

The cellular uptake of the developed SFB-loaded ME (ME16) was evaluated using a qualitative and quantitative manner using fluorescence microscopy and flowcytometry, respectively. Briefly, the breast cancer cells (4T1 cells) were seeded in a six-well plate with a density of 1 × 10^6^ cells/well and incubated at 37 °C overnight. The next day, coumarin-6 loaded ME formulation (Coumarin-6-ME16) was added to each well (n = 03), and cells were further incubated for another 6 h at 37 °C. Herein, the free coumarin-6 was added to the control group. After the incubation time, PBS was used to wash the cells at least three times, then fixed the cells with 4% paraformaldehyde solution for 15 min, and lastly, counterstained using DAPI. Further, cells were directly observed under a fluorescence microscope (Vert. A1 ZEISS Axiocam, Germany), and the images were acquired at excitation and emission of 488 nm and 510 nm, respectively. On the other hand, the cells were trypsinised, washed with PBS, and analysed using flowcytometry (Cytoflex, Beckman Coulter, USA) to quantify the cellular uptake of the coumarin-6-ME16. The flowcytometry data were processed and analysed using CytExpert software^36,37^.

### In-vivo pharmacokinetic studies

The pharmacokinetics were implemented on the Unisex Wistar rats (200–300 g; 4–6 weeks old). The *Institutional Animal Ethics Committee* (Panjab University, Chandigarh, India (PU/IAEC/S/16/18)) approved all the animal protocols. All experiments were performed under the relevant guidelines and regulations. The study in complied with Animal Research: Reporting of In-Vivo Experiments (ARRIVE) guidelines. The rats were separated into two different groups. The group I received free SFB suspended in 0.2% carboxymethyl cellulose, and group II administered the SFB-loaded ME via oral gavage. The human dose of SFB is 200 mg/70 kg which was applied to calculate the dose of SFB for each rat peroral route^38^. The blood samples (200 µL) were withdrawn at 0.25, 0.5, 1, 2, 4, 8, 12, 24 and 36 h time intervals from the retro-orbital plexus of rats. The plasma proteins were precipitated from the blood samples taken in heparin containing micro-centrifuge using micro-centrifugation at 12,000 rpm (10,866*g) for 10 min. Henceforth, the UHPLC method was incorporated to estimate SFB in the extracted plasma of rats at a wavelength of 265 nm with solvents (ACN and water; 65:35 v/v) and a flow rate of 0.8 mL/min using a C18 column^39^. Then, all the pharmacokinetic parameters such as Volume of distribution (V/F), maximum plasma concentration (C_max_), absorption rate constant (K_a_), time at the maximum concentration (T_max_), the area under the curve (AUC), half-life (t_1/2_) and elimination rate constant (K) were determined as per 1CBM peroral model using PK solver software^40^.

### Stability studies

The stability studies of the SFB-loaded ME were performed as per the guidelines of the International Council on Harmonization (ICH). To estimate the stability of the formulation, samples were stored under different storage conditions at 4 ± 2 °C, 30 ± 2 °C, 45 ± 2 °C for at least 6 months. The particle size, zeta potential and drug loading were observed at 0, 3rd and 6th months of interval^41^.

### Statistical analysis

Statistical analysis of the data was performed using *t*-test and analysis of variance (ANOVA) software. All the studies were performed at least three times (n = 3), and the data is shown as the means ± SD.

## Results and discussion

### Spectroscopic analysis

#### Fourier transform infrared spectroscopy (FT-IR)

**Figure 3:**
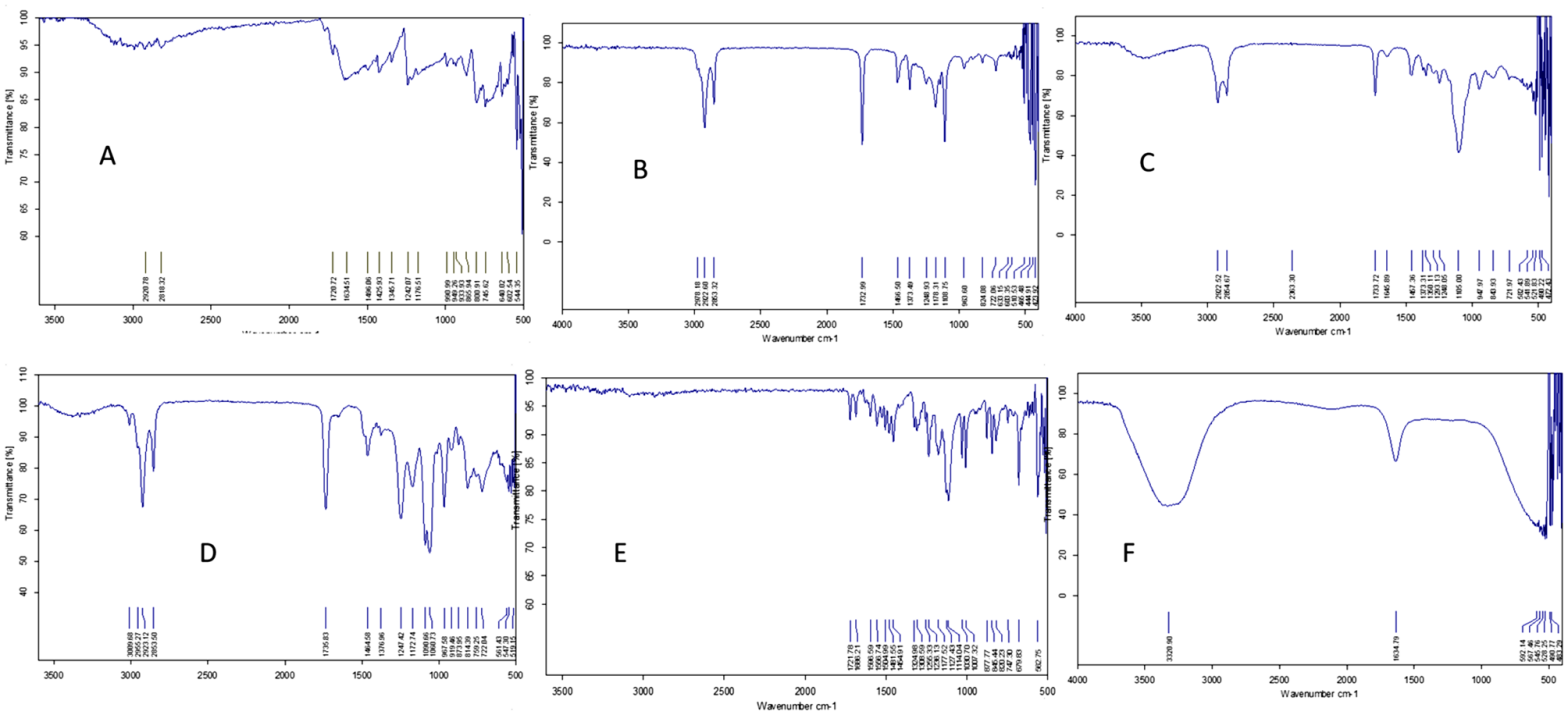
FTIR spectra of (**a**) Free SFB (**b**) Blank ME (**c**) SFB-loaded ME (**d**) Tween 80 (**e**) PL 90G (**f**) IPM.

The FT-IR spectra of SFB in Fig. 3A showed amide bond formation indicated peak at 2920.78 cm^−1^ ascribed presence of amine group in SFB. Various characteristic peaks were observed, in the range 1634.51–1425.93 cm^−1^. FT-IR spectra of blank ME (Fig. 3B), SFB-loaded ME (Fig. 3C), and excipients like Tween 80 (Fig. 3D) and PL (Fig. 3E), IPM (Fig. 3F) were also observed. The broadband centred at 3320.90 cm^−1^ was assigned to –OH stretching in PL. The absorption at 2978.18 cm^−1^ and 2922.60 cm^−1^ were assigned to –CH stretching of methylene group in blank ME. This absorption rate was decreased from 2922.52 to 2854.67 cm^−1^, significantly indicating the formation of SFB-loaded ME. There were broad spectra observed at 1106.00 cm^−1^ of the C–O stretch. Thus, it confirmed the formation of SFB-loaded ME^42^.

#### Ultra-high performance liquid chromatography (UHPLC)

**Figure 4:**
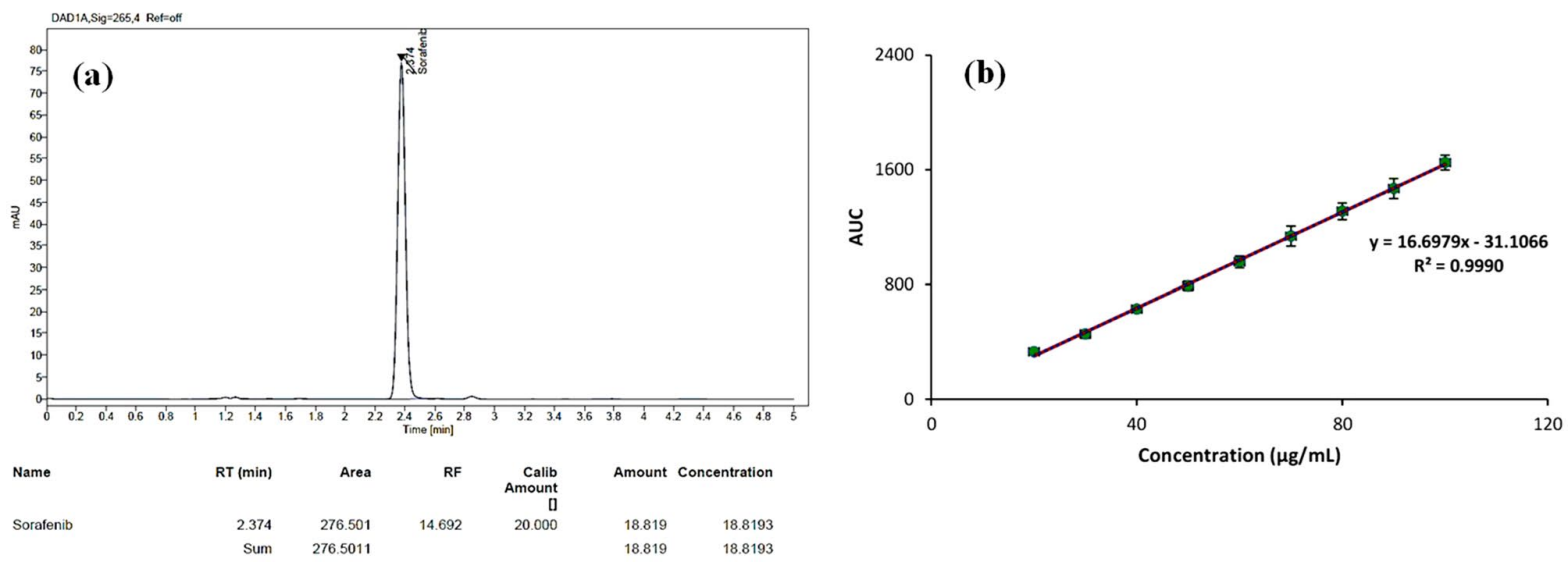
(**a**) Chromatogram of SFB. (**b**) Calibration curve of SFB.

The chromatogram and calibration curve of SFB using UHPLC has been demonstrated using ACN and water (65:35% v/v) as mobile phase under isocratic conditions with a flow rate of 0.8 mL/min and injection volume of 10 µL^39^. The Calibration curve and chromatogram are shown in Fig. 4a,b. It was observed that the retention time of SFB was determined to be 2.37 min, and r^2^ value was found to be 0.999.

#### Physicochemical characterization of SFB

The melting point of the drug was determined to be 204.5 °C, indicating the stability of SFB at higher temperatures, which was favourable for ME preparation. The practical log P value was estimated at 4.18, suggesting that the drug was highly lipophilic and suitable for oral delivery.

#### Screening of components of the microemulsion

To improve the aqueous solubility challenge of SFB, the IPM was selected as oil for ME preparation. In terms of toxicity, the nonionic surfactants show less toxicity in comparison to the ionic surfactants and have low critical micellar concentrations. SFB also showed solubility in these non-ionic surfactants. Therefore, four non-ionic surfactants were selected for the optimisation of ME^43^.

PL 90G and ethanol was selected as a cosurfactant for the ME in which PL 90G exhibited high biocompatibility and safety profile along with high solubility of SFB in ethanol. The selected ratio of PL 90G and ethanol was 1:10 to construct the phase diagram due to its maximum solubility with the drug and cost-effective nature. Solubility studies were performed in predetermined S_mix_ ratios i.e., 1:1, 2.75:1, 5:1, 10:1 and 20:1. Based on the outcomes of solubility studies, the S_mix_ ratio was chosen for the effective formulation development. The maximum solubility was manifested in the ratio 2.7:1 followed by 1:1 compared to other S_mix_ ratios.

#### Construction of pseudo-ternary phase diagram

**Figure 5:**
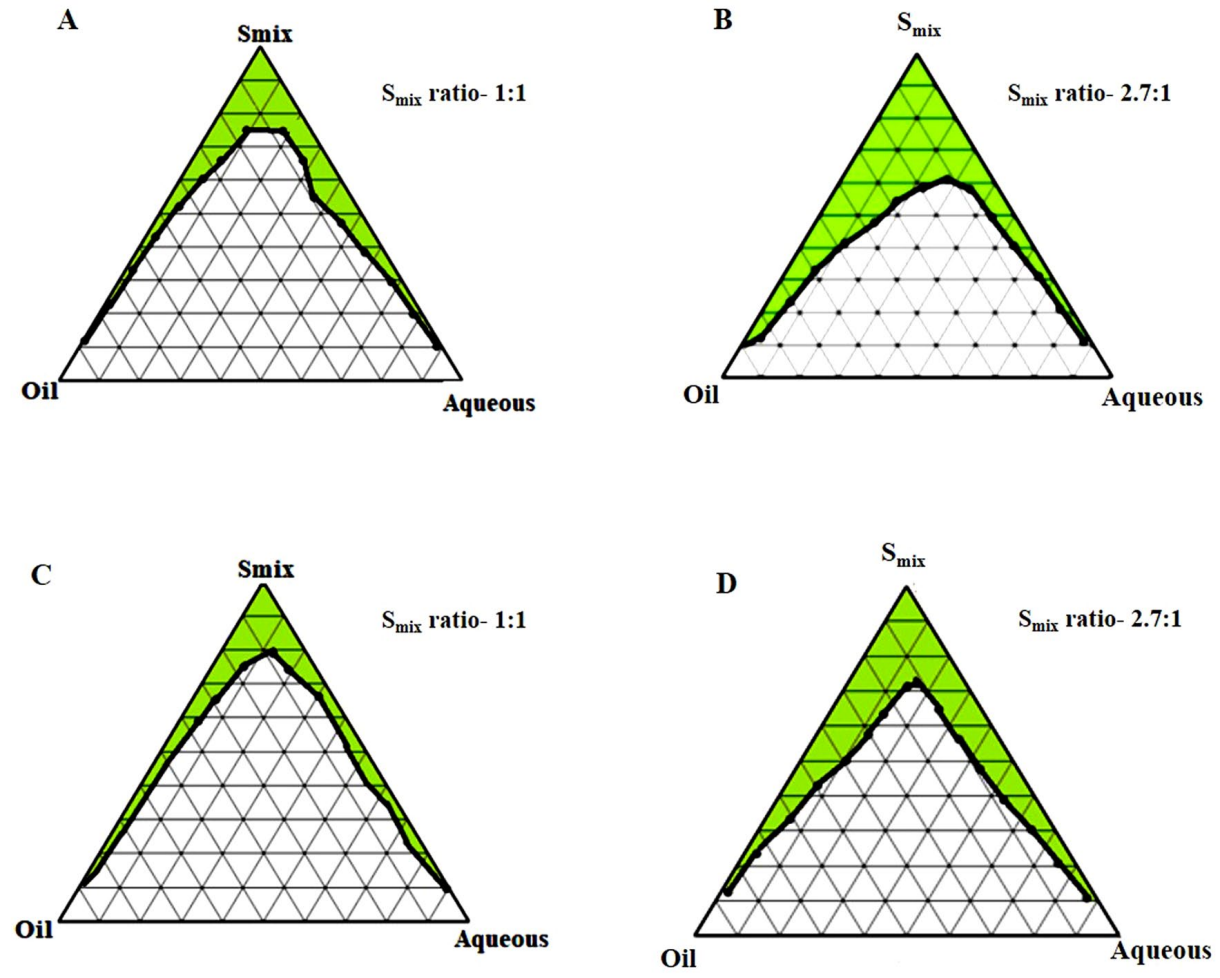
Pseudo-ternary phase diagrams; IPM as oil and T20 as a surfactant (**A** and **B**), T40 as a surfactant (**C** and **D**) at two S_mix_ ratios (1:1 and 2.7:1).

**Figure 6:**
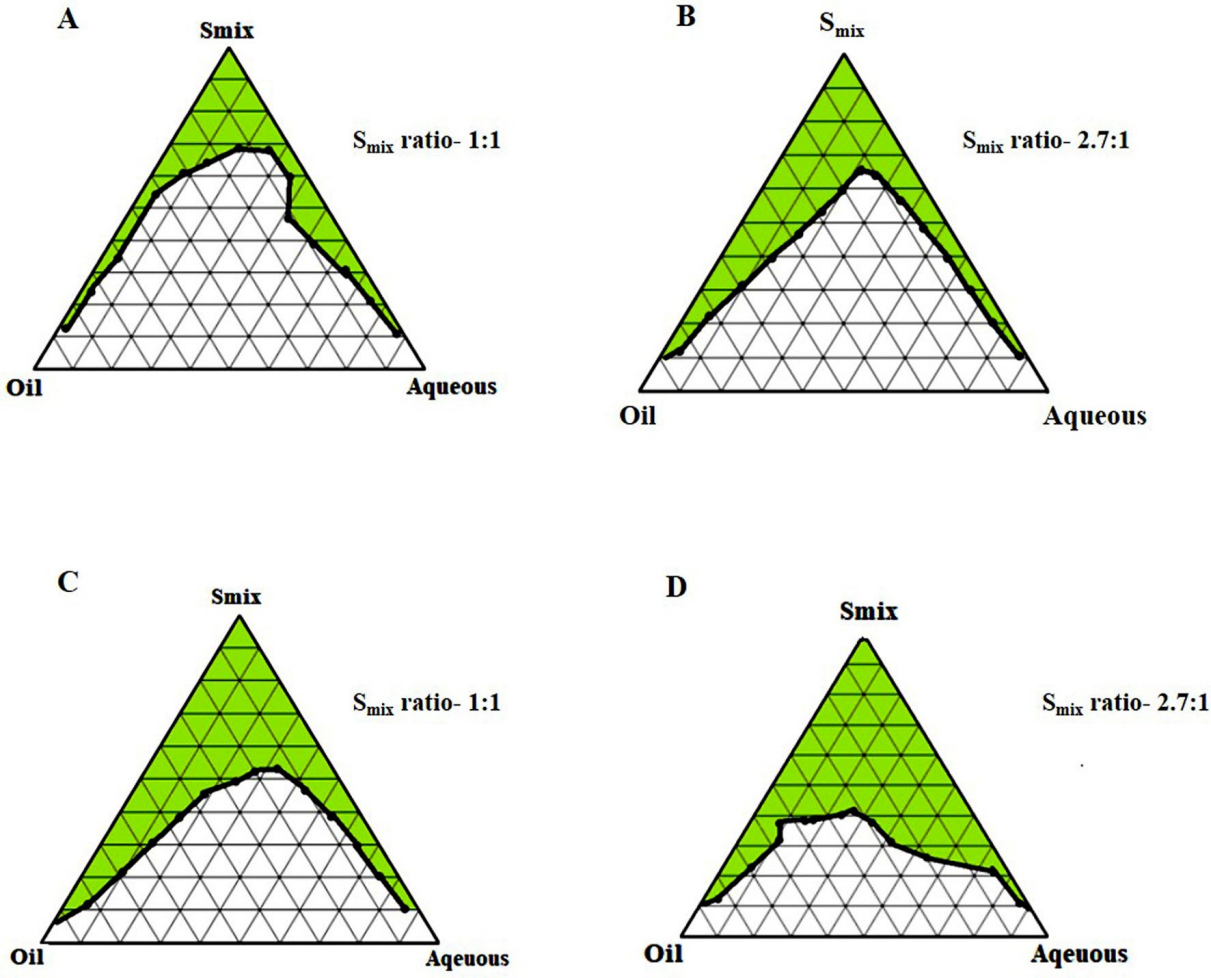
Pseudo-ternary phase diagrams; IPM as oil and T60 as a surfactant (**A** and **B**), T80 as a surfactant (**C** and **D**) at two S_mix_ ratios (1:1 and 2.7:1).

The phase behaviour of a mixture and its components relation can be easily identified by forming a phase diagram. The pseudo ternary phase diagrams of o/w ME of all the eight groups comprising IPM, ethanol, PL 90G, Tween 20/40/60/80 and distilled water are shown in Figs. 5 and 6. The ratio of surfactant and cosurfactant along with the oil played a significant role in enhancing the phase properties of the ME region. It can be observed in Fig. 5A,B that on increasing the concentration of surfactant (2.7:1), the ME area increased. A similar observation was also seen in other ternary phase diagrams. Also, the ME area was considerably smaller when T20, T40, and T60 were used as surfactants instead of T80, which offered a better emulsification region (32.45%). Hence, there were no miscibility issues of drug with the surfactants. The maximum emulsification property of T80 imposed its selection as the surfactant. Additionally, the loading of SFB was also assumed to be enhanced by the use of T80 as emulsifier. The stability of MEs was also enhanced by the presence of co-surfactant, which reduced the interfacial tension. The phospholipid 90G along with ethanol showed biocompatibility and reduced the phospholipid films rigidity needed for the ME globule formation^44^.

#### Selection of formulation based on phase diagram

Several MEs can be prepared from the ME region of a single-phase diagram. Due to the different surfactants used for the phase diagram construction, the number of formulations was also varied. From all eight pseudo-ternary phase diagrams, formulations were selected from each phase diagram. The composition of all of the selected sixteen ME formulations is given in Table 3.

**Table 3:** Composition of selected microemulsions.

Formulation code	Oil phase (%)	S_mix_ ratio	S_mix_ (%)
ME1	IPM (6%)	1:1	T20: (PL90G: ethanol) (88%)
ME2	IPM (10%)	1:1	T20: (PL90G: ethanol) (70%)
ME3	IPM (7%)	2.7:1	T20: (PL90G: ethanol) (70%)
ME4	IPM (2%)	2.7:1	T20: (PL90G: ethanol) (50%)
ME5	IPM (6%)	1:1	T40: (PL90G: ethanol) (40%)
ME6	IPM (3%)	1:1	T40: (PL90G: ethanol) (48%)
ME7	IPM (4%)	2.7:1	T40: (PL90G: ethanol) (46%)
ME8	IPM (10%)	2.7:1	T40: (PL90G: ethanol) (60%)
ME9	IPM (4%)	1:1	T60: (PL90G: ethanol) (56%)
ME10	IPM (8%)	1:1	T60: (PL90G: ethanol) (47%)
ME11	IPM (18%)	2.7:1	T60: (PL90G: ethanol) (66%)
ME12	IPM (4%)	2.7:1	T60: (PL90G: ethanol) (50%)
ME13	IPM (3%)	1:1	T80: (PL90G: ethanol) (74%)
ME14	IPM (2%)	1:1	T80: (PL90G: ethanol) (83%)
ME15	IPM (9%)	2.7:1	T80: (PL90G: ethanol) (69%)
ME16	IPM (9%)	2.7:1	T80: (PL90G: ethanol) (45%)

#### Drug content

All prepared formulations had a drug content of 99.42% to 99.83%, with an average value of 99.62% (Table 4). As the drug content was observed to be so high, it thereby confirmed minimal drug loss during the preparation of MEs and assured the authenticity of the preparation method.

**Table 4:** Characterization studies of microemulsion formulations.

Formulation code	Globule size (nm)	PDI	Zeta potential (mV)	Drug content (%)
ME1	1081.3 ± 0.4	0.69 ± 0.5	− 3.15 ± 0.6	99.49
ME2	862.1 ± 4.2	0.20 ± 0.03	− 0.22 ± 0.05	99.62
ME3	137.0 ± 3.8	0.32 ± 0.14	− 0.42 ± 0.21	99.68
ME4	513.5 ± 7.3	0.92 ± 0.11	0.31 ± 0.43	99.77
ME5	90.0 ± 6.6	0.23 ± 0.02	0.021 ± 0.15	99.53
ME6	122.6 ± 2.7	0.21 ± 0.03	− 0.36 ± 0.10	99.59
ME7	81.30 ± 0.49	0.21 ± 0.32	0.15 ± 0.07	99.67
ME8	79.09 ± 0.03	0.20 ± 0.05	0.16 ± 0.05	99.81
ME9	648.7 ± 1.02	0.57 ± 0.02	− 4.03 ± 0.02	99.46
ME10	512.8 ± 7.3	0.53 ± 0.51	− 1.67 ± 0.03	99.75
ME11	244.3 ± 0.5	0.65 ± 0.61	− 3.78 ± 0.12	99.62
ME12	83.2 ± 1.4	0.26 ± 0.14	− 2.11 ± 0.23	99.51
ME13	266.0 ± 2.5	0.65 ± 0.06	− 1.82 ± 0.04	99.42
ME14	319.3 ± 4.7	0.53 ± 0.05	− 2.57 ± 0.41	99.56
ME15	75.6 ± 0.2	0.20 ± 0.03	0.13 ± 0.11	99.71
ME16	58.8 ± 0.02	0.19 ± 0.14	0.05 ± 0.03	99.83

#### Micromeritics and zeta potential

**Figure 7:**
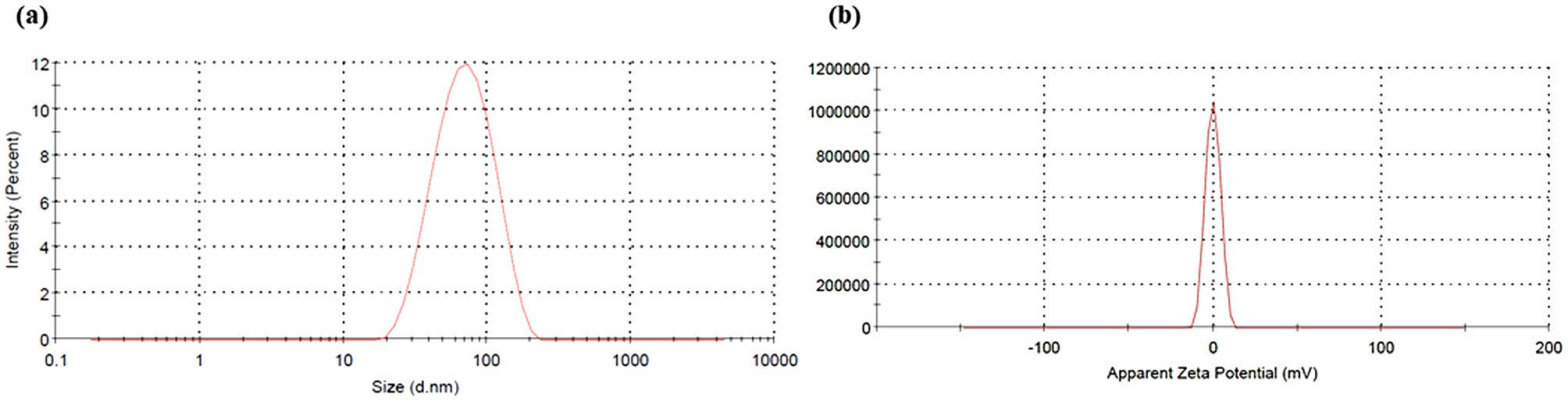
(**a**) Particle size of ME16 and (**b**) Zeta potential of ME16.

The zeta potential of several formulations was near to zero (Table 4), which attributed to the non-ionic nature of the surfactants used and imparted better stability to the MEs against any ionic reactions during the long-term storage. Out of all the formulations, the smallest globule size of the ME was of formulation ME16, i.e., 58.8 ± 0.02 nm (Fig. 7) with a zeta potential of 0.05 ± 0.03 (Table 4). The PDI of the ME16 formulation (0.19 ± 0.14) also showed the homogeneity of the ME system. Hence, these results ensured that ME16 was the optimized formulation and selected for further evaluation studies.

#### Percent drug loading (DL) and entrapment efficacy (EE) of ME

The percentage DL and EE of the selected SFB-loaded ME (ME16) was observed to be 21.07 ± 2.16% and 72.64 ± 0.84%, respectively. The value of EE depicted the higher amount of entrapment of SFB in ME with the help of an emulsifying agent (Tween 80). The drug-carrying capacity of the nano-system was quite favourable and assured high loading of SFB for better delivery to the target site.

#### Surface morphology

**Figure 8:**
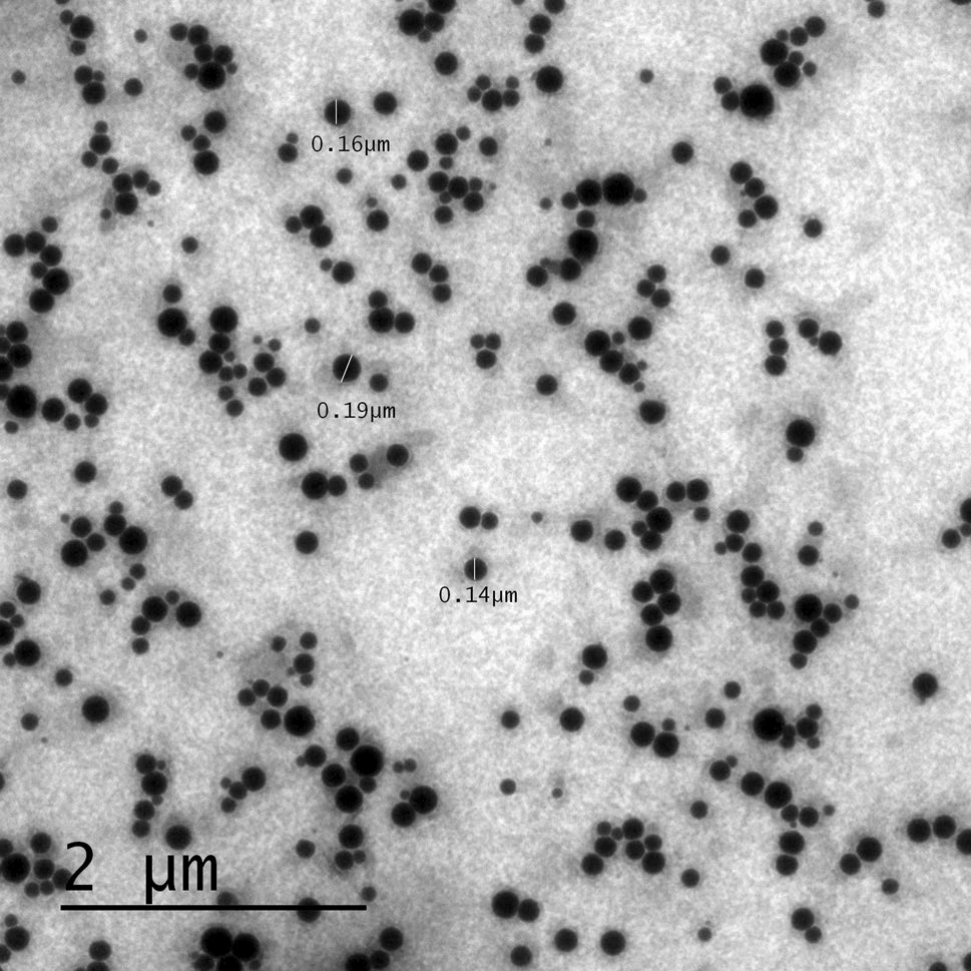
TEM image of SFB-loaded ME (ME16).

The photomicrographs (Fig. 8) of TEM exhibited a spherical shape of the ME16 globules with homogeneity in size. The TEM images showed the smooth surface of the developed ME deprived of any agglomeration^45^.

#### In-vitro drug release studies and release kinetics

**Figure 9:**
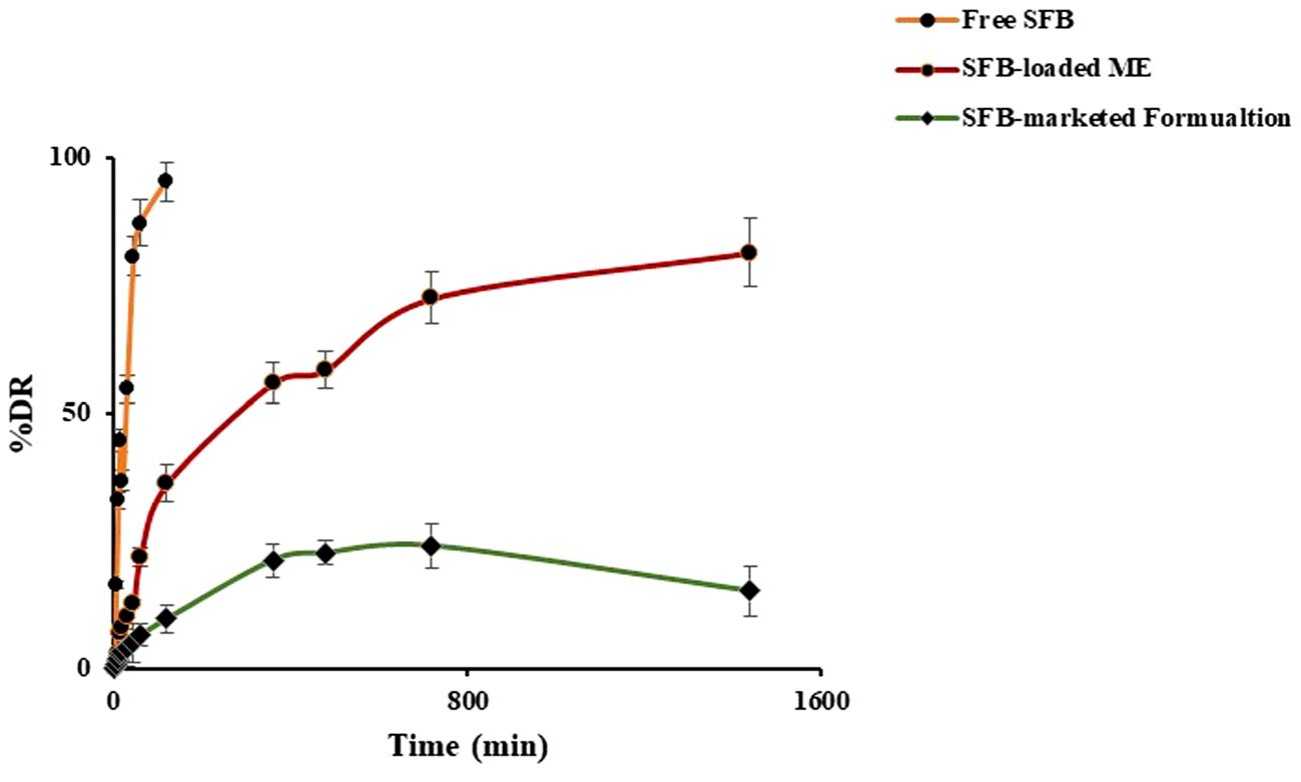
Graph of drug release study of free SFB, SFB-loaded ME (ME16) and SFB-marketed Formulation.

The drug release studies of free SFB, ME16 and SFB-marketed formulation (Soranib tablet, Batch no. GJ10330) was carried out in 0.1 N HCl for 2 h, followed by pH 6.8 for the further time period^46^. The graph (Fig. 9) depicted that the drug release was pH-dependent, and ME16 exhibited a sustained release pattern. Free SFB showed more than 90% of drug release within 2 h in simulated acidic medium (0.1 N HCl). The release of SFB from the marketed product across the dialysis membrane was quite less as shown in the Fig. 9. On the other hand, the drug dissolution profile of the marketed product (Soranib tablet, Batch no. GJ10330) using the USP II apparatus following a USFDA approved protocol^47^ was well within the acceptable range (Supplementary data, Fig. S1). The reason for slow release by the marketed product might be the presence of dialysis membrane. The developed microemulsion system was able to sustain the drug release over 24 h, however, the drug dissolution of marketed product released the drug in 1 h. The results unequivocally vouch the sustained and controlled drug release behavior of the developed system. On fitting the values of % drug release versus time into the various release kinetic models, it was inferred that ME16 followed the Higuchi kinetic model. The r^2^ values of multiple models are depicted in Table 5^48^.

**Table 5:** Parameters of various release kinetic models.

Release kinetic models	Formulations	Parameters		
		Slope	Intercept	r^2^
Zero order	Pure drug	0.0296	18.769	0.4996
	SFB-loaded ME	0.055	20.834	0.662
	SFB-marketed formulation	0.0124	4.9346	0.4992
First order	Pure drug	0.007	1.067	0.256
	SFB-loaded ME	0.0009	1.07	0.334
	SFB-marketed formulation	0.0007	0.5248	0.4308
Higuchi	Pure drug	0.565	3.004	0.754
	SFB-loaded ME	0.379	1.361	0.889
	SFB-marketed formulation	1.3215	0.8616	0.7364

#### Protein binding studies

**Figure 10:**
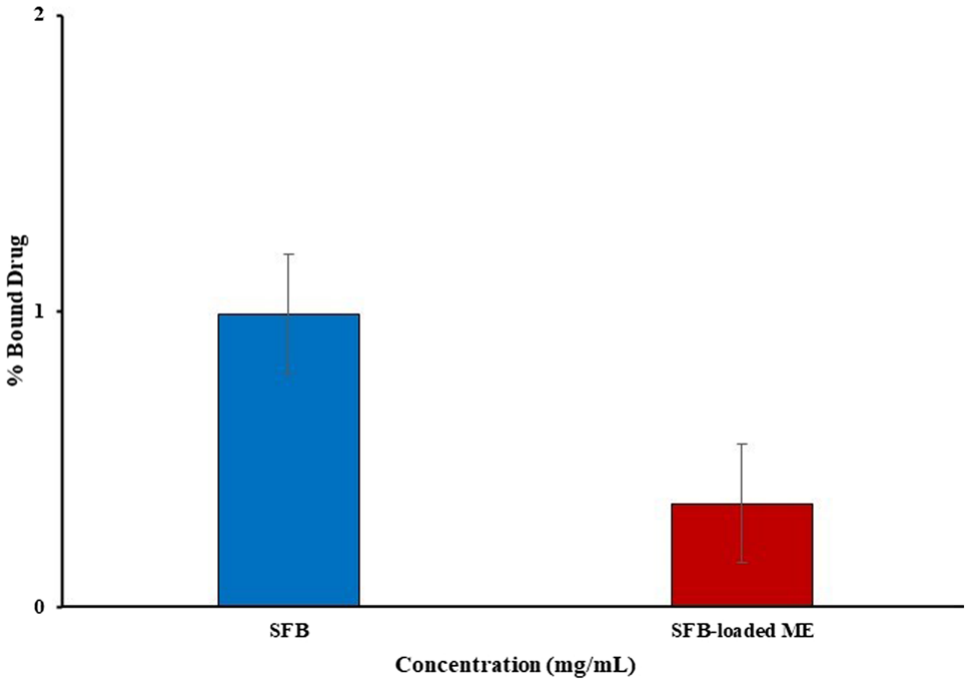
Graph of protein binding of free SFB and SFB-loaded ME (ME16).

Protein binding studies in the equivalent doses of 10 mg/kg for the free SFB at a specific 1 mg/mL concentration is > 90%^49^. The study revealed that after loading onto the ME, the protein binding was substantially decreased (p < 0.05), as shown in Fig. 10. The protein binding of free SFB was more than the ME16. It stated that the developed formulation could provide better affinity of SFB to the target site as compared to the free SFB.

#### In-vitro cytotoxicity assay

**Figure 11:**
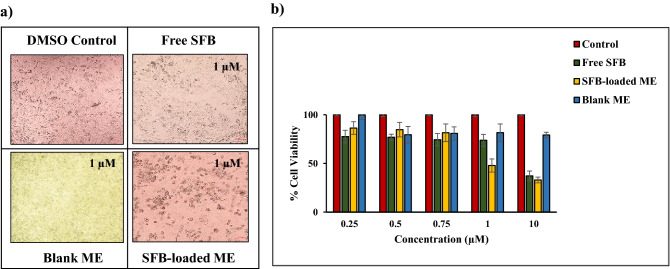
Cytotoxicity evaluation of SFB-loaded ME concerning free SFB determined via (**a**) microscopic evaluation and (**b**) MTT based cell viability assay.

The cytotoxicity of SFB, blank ME, and SFB-loaded ME against 4T1 breast cancer cells were evaluated through microscopic observations and IC_50_ value determination. The microscopic images of the treated 4T1 cells were captured at 1 μM concentration, considering DMSO as control (Fig. 11a)^50^. Through these microscopic observations, blank ME exhibited no cytotoxic effect on 4T1 breast cancer cells. A significant decrease in cell viability was observed in the case of SFB-loaded ME than free SFB at 1 µM concentration. The cytotoxic effects of different samples are also represented as % cell viability versus concentration plot in the Fig. 11b). Though at lower concentration, the efficacy of both free SFB and SFB-loaded ME are comparable, but at the concentration above 1 µM, the difference is conspicuous (p < 0.05). The IC_50_ values of free SFB and SFB-loaded ME were calculated to be 3.47 ± 0.21 and 2.15 ± 0.11 µM, respectively. These outcomes confirmed the therapeutic efficacy of the drug loaded ME. Considering the IC_50_ values, the lethal dose 50 (LD_50_) values of free SFB and SFB-loaded ME were calculated to be 8.99 and 7.52 µM, respectively. The large therapeutic window provided by SFB-loaded ME supported the safety profile of the formulation. The findings are of greater significance as the dose of the drug for human is 200 mg and the plasma drug concentration for 100% bioavailability (theoretically) comes out to be 350 ng/mL. It implies that 0.136% of the centrally available drug concentration has the potential to kill the cancer cells, when loaded in the developed ME.

#### In-vitro cellular uptake study

**Figure 12:**
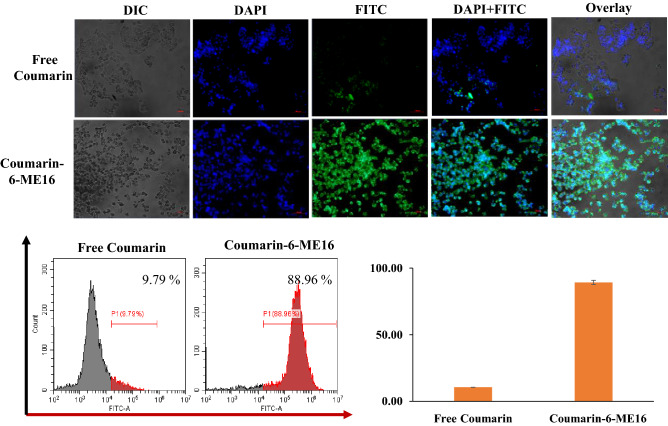
Cellular uptake study of free coumarin and coumarin-6-ME16.

The cellular uptake study was performed and analysed qualitatively and quantitatively on 4T1 breast cancer cells. For the qualitative determination of cellular uptake, coumarin-6-ME16 and free coumarin was observed using a fluorescence microscope (Zeiss, Germany). As shown in Fig. 12**,** the results revealed higher cellular internalization in case of coumarin-6-ME16 in overlay images. On the other hand, free coumarin did not exhibit significant cellular uptake through microscopic observation. To further confirm the qualitative results, the uptake was quantified using flowcytometry (Cytoflex, Beckman Coulter, USA). The amount of coumarin-6 internalised by the 4T1 cells was assumed to impart the fluorescence intensity. The findings obtained from CytExpert software showed that 9.79% of free coumarin was internalized by the cells, whereas the uptake was 88.96% in the case of coumarin-6-ME16. Qualitative and quantitative analysis confirmed higher 4T1 cell uptake in the case of coumarin-6-ME16, which also supported the cytotoxicity results.

#### In-vivo pharmacokinetic studies

**Figure 13:**
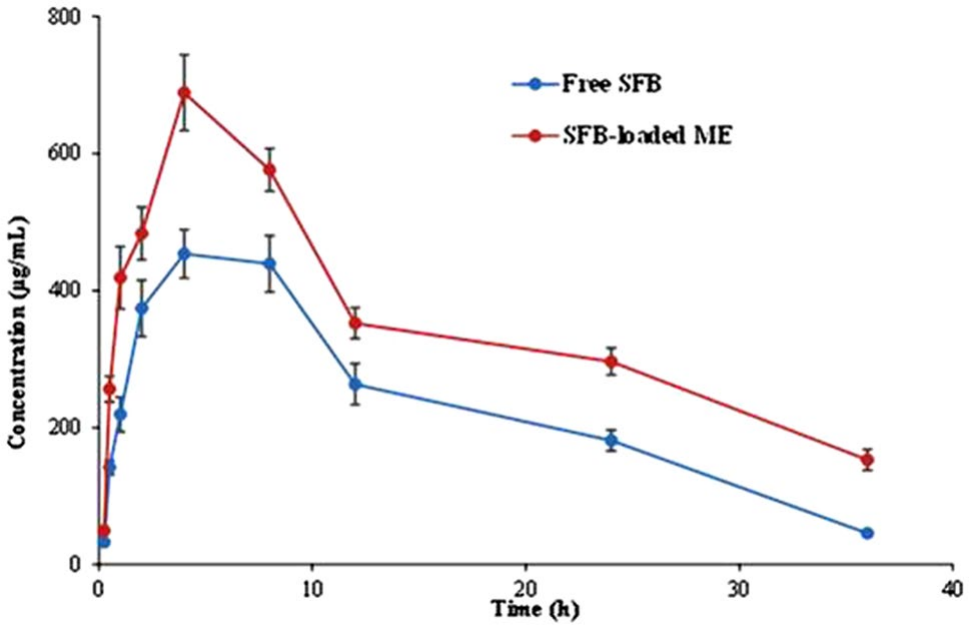
Plasma-concentration time graph of Free SFB and SFB-loaded ME (ME16).

The free SFB and SFB-loaded ME was evaluated for further in-vivo pharmacokinetic studies. The study’s graph was plotted between the plasma drug concentration versus time, as shown in Fig. 13. The graph estimated that the rats of group II (ME16) exhibited a higher concentration of plasma at each time point than of the group I receiving free SFB (p < 0.05). The pharmacokinetic parameters were determined by fitting the plasma concentration–time profile in PK solver software. A comparison of various pharmacokinetic parameters has been described in Table 6. The mean residence time (MRT) value of ME16 (21.82 h) was found to be significantly higher than the free SFB (16.49 h; p < 0.05). This result revealed a reduction in the clearance rate of the drug. The clearance rate (CL) of the free drug was found to be 0.09 μg/ml compared to the SFB-loaded ME (0.05 μg/ml). The high entrapment of SFB into the ME could be justified because of the predicted low level of volume of distribution of ME16 (1.08 μg/ml). It is evident from the value of pharmacokinetic parameters that fabrication of SFB in the form of ME resulted in the formulation with a sustained effect. The T_max_ was almost safe. However, the bioavailability of SFB was 1.5 times increased by the ME system. The findings provide hope for a formulation with the potential to enhance the biological stay period of the drug and increase the bioavailability fraction. It has immense promises in better efficacy and dose reduction.

**Table 6:** Various pharmacokinetic parameters of ME16 and free SFB.

Parameters	Unit	SFB-loaded ME	Free SFB
V/F	(mg)/(μg/ml)	1.08	1.30
CL/F	(mg)/(μg/ml)/h	0.05	0.09
T_max_	h	4.02	4.70
C_max_	μg/ml	625.31	456.47
AUC 0-t	μg/ml h	12,686.98	8238.03
AUC 0-inf	μg/ml h	15,550.59	9113.58
MRT	h	21.82	16.49

#### Stability studies

The stability studies of the developed ME16 were carried out for 6 months^51^; It was observed that 30 ± 2 °C was the most favourable storage conditions for the SFB-loaded ME as no significant changes in particle size, zeta potential and drug loading had been found in this temperature (Table 7).

**Table 7:** Stability studies of SFB-loaded ME (ME16) at different temperature conditions.

Parameters	Particle size (nm)	Zeta potential (mV)	Drug loading (%)
Months	4 ± 2 °C		
0	55.2 ± 0.13	− 0.03 ± 0.09	22.07 ± 1.18
3rd	65.8 ± 0.04	− 0.12 ± 0.31	21.41 ± 0.32
6th	56.2 ± 0.02	− 0.21 ± 0.37	23.07 ± 1.8

	30 ± 2 °C		
0	54.2 ± 0.04	0.05 ± 0.08	21.07 ± 2.16
3rd	56.3 ± 0.06	0.06 ± 0.02	21.04 ± 1.86
6th	56.8 ± 0.02	0.05 ± 0.05	21.14 + 0.96

	45 ± 2 °C		
0	55.8 ± 1.02	0.06 ± 0.04	21.12 ± 2.01
3rd	57.1 ± 1.89	0.08 ± 1.3	19.02 ± 0.04
6th	59.07 ± 0.34	0.10 ± 0.35	19.32 ± 1.87

## Conclusion

The challenges of SFB related to oral delivery are limiting its efficacy towards breast cancer. The present reports, i.e., in-vitro and in-vivo studies, manifested a new insight towards the SFB oral administration by developing SFB-loaded ME. The developed formulation enhanced the oral bioavailability of the SFB and increased its t1/2 which could result in a once-in-a-day product. The overall performance and cytotoxicity of the SFB to the breast cancer cells were improved after encapsulated into the ME as depicted in cell viability studies. The same can be further investigated in the preclinical studies for confirmation and further exploration.

## Supplementary Information

Supplementary Figure S1.

## Data Availability

The data used to contribute the findings of this research are included within the article and supplementary file.
