# Supplementary material for: Oral sorafenib-loaded microemulsion for breast cancer: evidences from the in-vitro evaluations and pharmacokinetic studies
Source: Sci Rep. 2022 Aug 12;12:13746. doi: 10.1038/s41598-022-17333-6 (PMC9374710; doi:10.1038/s41598-022-17333-6)
Supplement: Supplementary file 1 — Supplementary Figure S1. [file 41598_2022_17333_MOESM1_ESM.docx]

**Supplementary Information**

**Oral Sorafenib-loaded Microemulsion for Breast Cancer: Evidences from the *In-vitro* Evaluations and Pharmacokinetic Studies**

Nishtha Chaurawal^1^, Charu Misra^1^, Teenu Sharma^2,3^, Reena Jatyan^4^, Deepak Chitkara^4^, Md. Abul Barkat^5^, Harshita Abul Barkat^5^, Bhupinder Singh^2^, Kaisar Raza^1,^*

^1^Department of Pharmacy, School of Chemical Sciences and Pharmacy, Central University of Rajasthan, Bandarsindri, Ajmer, Rajasthan, India-305817.

^2^University Institute of Pharmaceutical Sciences, Panjab University, Chandigarh, India-160014.

^3^Department of Pharmacy, Chandigarh College of Pharmacy, Landran, Punjab, India-140307.

^4^Department of Pharmacy, Birla Institute of Technology and Science (BITS)-Pilani, Pilani Campus, Pilani, Vidya Vihar, Rajasthan, India-333031.

^5^Department of Pharmaceutics, College of Pharmacy, University of Hafr Al-Batin, Al Jamiah, Hafr Al Batin, 39524, Saudi Arabia.

**Dissolution Test.** The dissolution study of SFB-marketed formulation (Soranib tablet, Batch no. GJ10330) was performed using dissolution test apparatus (USP apparatus type II paddle), following a reported protocol of USFDA.^1^ As per the methodology, 0.1 M HCl with 1% SDS was preferred as the dissolution media and the rotation speed was maintained at 75 rpm. Samples (2 mL) were withdrawn after pre-determined time intervals (0, 5, 10, 15, 20, 30, and 60 min) and the sink was maintained with fresh media. The results revealed that approximately 75% of drug was released into the media within 15 min. However, almost 90% of the drug was released into the media within 60 min **(Figure S1)**. The SFB-marketed formulation exhibited immediate drug release in the dissolution test.

**Figure S1:** Graph of dissolution study of SFB-marketed Formulation.
